# Robotic image-guided reirradiation of lateral pelvic recurrences: preliminary results

**DOI:** 10.1186/1748-717X-6-77

**Published:** 2011-06-23

**Authors:** Sylvain Dewas, Jean Emmanuel Bibault, Xavier Mirabel, Philippe Nickers, Bernard Castelain, Thomas Lacornerie, Hajer Jarraya, Eric Lartigau

**Affiliations:** 1 Département Universitaire de Radiothérapie, CyberKnife Nord-Ouest, Centre Oscar Lambret, CLCC, Université Lille II, Lille, France; 2 Département d'Oncologie Digestive, Centre Oscar Lambret, CLCC, Lille, France; 3 Département d'Oncologie Gynécologique, Centre Oscar Lambret, CLCC, Lille, France; 4 Département d'imagerie Médicale, Centre Oscar Lambret, CLCC, Lille, France

## Abstract

**Background:**

The first-line treatment of a pelvic recurrence in a previously irradiated area is surgery. Unfortunately, few patients are deemed operable, often due to the location of the recurrence, usually too close to the iliac vessels, or the associated surgical morbidity. The objective of this study is to test the viability of robotic image-guided radiotherapy as an alternative treatment in inoperable cases.

**Methods:**

Sixteen patients previously treated with radiotherapy were reirradiated with CyberKnife^® ^for lateral pelvic lesions. Recurrences of primary rectal cancer (4 patients), anal canal (6), uterine cervix cancer (4), endometrial cancer (1), and bladder carcinoma (1) were treated. The median dose of the previous treatment was 45 Gy (EqD2 range: 20 to 96 Gy). A total dose of 36 Gy in six fractions was delivered with the CyberKnife over three weeks. The responses were evaluated according to RECIST criteria.

**Results:**

Median follow-up was 10.6 months (1.9 to 20.5 months). The actuarial local control rate was 51.4% at one year. Median disease-free survival was 8.3 months after CyberKnife treatment. The actuarial one-year survival rate was 46%. Acute tolerance was limited to digestive grade 1 and 2 toxicities.

**Conclusions:**

Robotic stereotactic radiotherapy can offer a short and well-tolerated treatment for lateral pelvic recurrences in previously irradiated areas in patients otherwise not treatable. Efficacy and toxicity need to be evaluated over the long term, but initial results are encouraging.

## Background

Cancers such as prostate adenocarcinoma, epidermoid carcinoma of the uterine cervix, and adenocarcinoma of the rectum receive pelvic radiotherapy as part of their initial treatment. Locoregional recurrence occurs in 3% to 15% of patients treated for rectal adenocarcinoma [1] and 1.5% to 40% of patients treated for carcinoma of the uterine cervix [2]. Better systematic monitoring of these pathologies, as well as progress in imaging, enabled earlier diagnosis of locoregional pelvic recurrences. However, in cases of lateral pelvic recurrence, therapeutic options are often limited. In these situations, surgery is often proposed, but unfortunately, few patients are found eligible because of the lateral location, the proximity of the iliac vessels and the associated surgical morbidity. Traditionally, an invasion of the lateral pelvic wall and/or envelopment of the iliac vessels are contraindications to a local radical procedure [3]. Without treatment, these patients have a short life expectancy and tend to experience symptoms, especially pain, with their quality of life becoming extremely poor [4].

Recent progress in image-guided radiotherapy (IGRT) has allowed a significant increase in the dose to the tumor volume while decreasing the dose to the neighboring organs at risk. Since June 2007, a robotic IGRT system, the CyberKnife^® ^(Accuray Incorporated, Sunnyvale, California, USA) has been available at the Centre Oscar Lambret in Lille, France. This system is capable of delivering extracranial stereotactic radiotherapy with millimetric precision [5-7]. Here, we report the tolerance and feasibility of robotic IGRT for lateral pelvic recurrences based on our preliminary experience in 16 patients reirradiated with CyberKnife.

## Methods

### Patients

Sixteen patients have been reirradiated with the CyberKnife for lateral pelvic lesions at our center since June 2007. The primary diseases were six anal canal lesions, four rectal cancers, four uterine cervical cancers, one endometrial cancer, and one bladder carcinoma. Ten women and six men were treated. The mean age at the recurrence was 55 years (range, 35 to 70 years). These patients had previously received, as part of their initial treatment, pelvic radiotherapy in the form of external-beam radiotherapy or brachytherapy. The median prior dose was 45 Gy (range, 52 to 96 Gy). Patient characteristics and biologically equivalent doses received by the lateral pelvic wall are reported in Table 1.

**Table 1:** Characteristics of patients treated with CyberKnife for pelvic re-irradiation.

	Number (%)	Mean (range)	Comments
Patients	16		
Sex (M/F)	6 (37%)/10 (63%)		
Age*	55	(34 - 70 y.o.)	

Primary disease			
Anal canal	6 (38%)		
Cervix	4 (25%)		
Uterus	1 (6%)		
Rectum	4 (25%)		
Bladder	1 (6%)		

Primary treatment			
Surgery	9 (56%)		
Chemotherapy	13 (81%)		9 concomitant; 4 adjuvant
Radiotherapy	14 (87%)		
Dose*		45 Gy (20-75 Gy)	
Eq D2*			
Early side effects (α/β = 3 Gy)		45 Gy (33-58 Gy)	
Late side effects (α/β = 10 Gy)		72 Gy (53-96 Gy)	

Treatment of the recurrence			
Surgery	6 (38%)		
Chemotherapy	8 (50%)		
Radiotherapy	3 (19%)		
Dose*		53.7 Gy (36-66 Gy)	
Eq D2*			
Early side effects (α/β = 3 Gy)		65 Gy (45-66 Gy)	
Late side effects (α/β = 10 Gy)		106 Gy (72-110 Gy)	

* Median value

### Lateral pelvic recurrence

**Figure 1:**
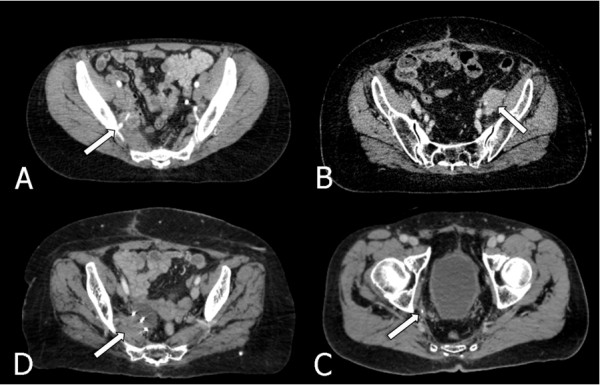
**Examples of pelvic recurrence in previously irradiated areas**: (A) Rectal cancer recurrence near the right iliac vessels (B) Cervix cancer recurrence near the left iliac vessels (C) Right pelvic anal canal recurrence (D) Rectal cancer recurrence previously (3 surgical clips visible).

Pelvic recurrence was discovered during systematic imaging examination for thirteen patients (example shown in Figure 1) and by clinical symptoms such as pain for three others. To rule out distant metastases, thoracic-abdominal-pelvic CT and FDG-PET were performed before CyberKnife treatment. The median disease-free interval between initial treatment and recurrence was 27 months (range, 4 to 148 months). All referrals for CyberKnife treatment were reviewed by a multidisciplinary board. One patient presented with bilateral pelvic recurrence from cervical cancer. Eight patients had chemotherapy before stereotactic radiotherapy (three to 10 courses of chemotherapy). Six patients had local surgical excisions. Five excisions were interrupted because of difficulties with neurovascular dissection and were consequently classified R2 (incomplete macroscopic resection). Three patients received radiation therapy (mean 53.7 Gy, 36 to 66 Gy) to the pelvis as part of the treatment for recurrence before CyberKnife treatment.

### CyberKnife treatment

**Figure 2:**
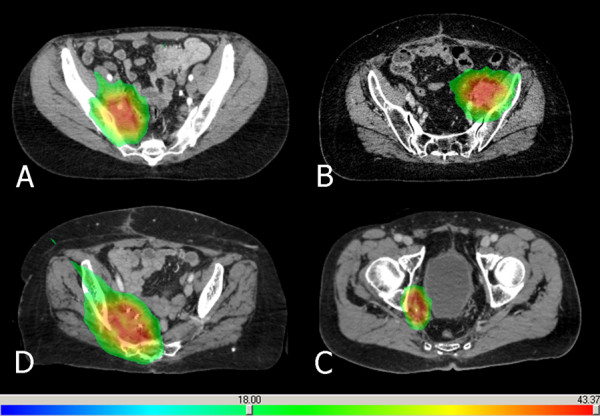
**Dosimetry for pelvic stereotactic radiotherapy by CyberKnife for each patients presented in figure 1**. Prescription to the 80% isodose line covering 95% of the PTV.

A vacuum mattress was used for immobilization during imaging and throughout treatment. Contrast agent was used for the planning CT. The gross tumor volume (GTV) was contoured on a fused CT/MRI matrix. The clinical target volume (CTV) was equal to GTV. The planning, set-up and treatment were conducted using Xsight^® ^Spine (Accuray) tracking method available with the CyberKnife system. Patient positioning and image guidance was performed with Accuray's Xsight Spine algorithm with registration to the patient's spine and pelvic bones. The median size of the tumor targets was 34.5 mm (range, 14 to 50 mm). Planning target volume included the CTV and a 3-mm margin due to the distance of the target from anatomical landmarks used for the tracking process and the uncertainty that might entail [8]. The treatment delivered a total dose of 36 Gy in six fractions over three weeks with a 6 MeV beam. The dose was prescribed to the 80% isodose line covering 95% of the PTV (Figure 2). Dose calculation was performed using the Ray-Tracing algorithm. One patient received concomitant Cetuximab combined with a platinum salt. One patient received 45 Gy in 3 fractions, considering the location of the lesion, away from the iliac vessels.

### Follow-up and statistics

This was a single-institution retrospective study conducted at the Centre Oscar Lambret. All subjects signed an informed consent form prior to treatments. The patients were followed systematically every 3 months after CyberKnife treatment for a median follow-up of 10.6 months (1.9 to 20.5 months). Follow-up visits included both clinical and laboratory tests. CTCAE v3.0 was used for scoring GI and GU toxicity. The responses were evaluated according to RECIST v1.1 criteria on CT-Scan performed every 3 months. The endpoints studied were treatment feasibility, toxicity and preliminary local control. Quality of life improvements based on patient self-reports and pain relief based on discontinuation of opiate usage were also examined. Local control, overall survival and disease-free survival analysis were carried out using the Kaplan-Meier method. Statistical packages SPSS 13.0 (SPSS Inc., Chicago, IL, USA) were used to perform the analysis.

## Results

### Feasibility

All patients were in excellent general condition at the time of recurrence and CyberKnife treatment (WHO = 0/1 for 15 patients and WHO = 2 for one). Eight patients described a sciatic pain at the time of diagnosis of the recurrence. All these patients were taking opioid pain medication before beginning irradiation. The median interval between the diagnosis of the recurrence and the beginning of the CyberKnife treatment was 5.3 months (range, 1 to 31 months). All treatments were delivered uneventfully as planned. The mean duration of the sessions was 48 minutes (range, 32 to 75 min) for the sessions of 6 Gy and 78 min for the patient treated in three 15-Gy sessions.

### Efficacy

**Figure 3:**
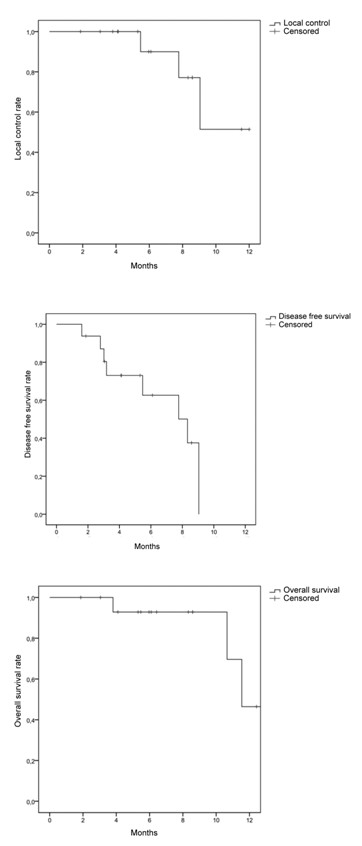
**The overall median survival after CyberKnife treatment was 11.5 months and 25.7 months after diagnosis of the pelvic recurrence**. The actuarial one-year survival rate was 46%. The one-year actuarial local control rate was 51.4%. Median disease-free survival was 8.3 months after CyberKnife treatment.

Median follow-up was 10.6 months (1.9 to 20.5 months). The overall median survival after CyberKnife treatment was 11.5 months and 25.7 months after diagnosis of the pelvic recurrence. The actuarial one-year survival rate was 46%. The one-year local control rate was 51.4%. Treatment of adenocarcinoma tended to result in better local control than squamous carcinoma (p = 0.09). The time between initial disease and recurrence and between recurrence and CyberKnife treatment did not modify overall survival or local control. Median disease-free survival was 8.3 months after CyberKnife treatment. Disease-free survival rate at 6 months was 63% (95% IC: 49 to 77%) (Figure 3). Four of the eight patients (50%) with pain described an improvement in terms of pain relief at the end of radiotherapy. However discontinuation of opioid treatment was never possible following the treatment.

### Toxicity

Acute toxicity was limited to grade 1 and 2 complications. One patient (6.25%) presented with nausea/vomiting (grade 2), two patients (12.5%) with diarrhea (grade 1) at the end of treatment and one patient (6.25%) had an increase in pain (grade 1). One patient (6.25%) experienced grade 2 digestive toxicity 3 months post-treatment. A grade 2 edema on the ipsilateral leg was described in one patient (6.25%) 3 months after treatment. At six months, a patient (6.25%) presented with grade 2 anorexia. To the date of this publication, no grade 3 or grade 4 toxicity has been observed.

## Discussion

We report a series of 16 patients treated for lateral pelvic recurrences in the proximity of iliac vessels. The one-year local control rate was 51.4% according to RECIST criteria with a median follow-up of 10.6 months. The overall median survival after CyberKnife treatment was 11.5 months and 25.7 months after diagnosis of the pelvic recurrence. It seemed that the treatment delayed local disease progression in all cases considering the rapid rate of progression commonly observed with these lesions [9,10]. In addition, it provided quality of life benefits by improving pain control in four patients based on self-reports. Tolerance of the technique was excellent. No grade 3 or 4 toxicity was observed. The toxicities described were essentially of digestive nature with one anorexia, some nausea and vomiting. There were no persistent urinary or gastrointestinal toxicities observed. Overall the feasibility of this technique was good.

Our population was not homogenous in regard to the histology of the primary lesion. Despite this variation, the treatment challenges presented by lateral pelvic recurrences in a previously irradiated area in this cohort were similar. The chance of survival also depended on the site of the recurrence and the stage of the disease at diagnosis rather than the pathology of the primary. Lateral pelvic surgery is often difficult and highly risky. Operations often have to be prematurely aborted, resulting in a type R2 resection (incomplete macroscopic resection) because of the proximity of the iliac vessels. In our series, surgical excision was attempted and failed to reach completion in five patients due to difficulties posed by vascular dissection.

Suzuki et al. have reported that none of the patients with recurrent rectal cancer who have undergone surgical excision survived even five years because of the impossibility of applying sufficiently generous margins [11]. Complete surgical removal may be the only hope for long-term survival, yet comes with the price of significant morbidity [12,13]. Curative surgical approach can only be achieved when margins are negative for microscopic extension of disease. In case of recurrences of rectal adenocarcinoma, this can be achieved in about 45% of cases, ranging from 10% to 67% in the published literature. Thus, resection of local recurrence usually requires major surgery, involving removal of adjacent pelvic organs. Operative morbidity varies from 22% to 100% [3]. Surgical treatment of pelvic recurrence in the uterine cervix has also been reported. Surgical salvage is feasible in only a small number of central recurrences and that too involves all the associated morbidity and mortality. Surgery, in most of these patients, amounts to either anterior, posterior or total exenteration [9]. The procedure is thus highly morbid and results in severe quality of life challenges. In short, treatment remains a challenge and no consensus exists as to an optimal treatment approach [10].

Consequently, pelvic stereotactic radiotherapy represents an attractive alternative for patients otherwise not treatable. Robotic IGRT is now equipped with technology capable of delivering ablative doses of radiation to clinical targets with high conformality and homogeneity. This may result in a more durable response of pelvic recurrences in patients who have already received a dose of pelvic irradiation and whose tumors are not amenable to surgery.

There are several studies reporting on SBRT for pelvic reirradiation for recurrent gynaecological cancer [14] and recurrent rectal cancer [15]. Pelvic stereotactic re-irradiation has been reported in the literature in an article on treatment with CyberKnife of 23 patients with recurrent rectal carcinoma four of whom had received prior radiation [16]. In that report, which was a dose escalation study, 16 of the recurrences were localized in the lateral pelvic wall. Fiducial markers were employed in the image-guided tracking of the targets. A geometric expansion of 3 mm was applied to the GTV in all directions to obtain the PTV and the doses delivered ranged from 16 Gy to 51 Gy. The five-year survival rate was 23.2% and overall median survival 37 months. The rate of survival without progression at four years was 74.3%. They reported a single grade-4 toxicity, a digestive track perforation, in a patient who received 51 Gy in three fractions. No specific toxicity was reported in the four previously treated patients. A second study reported by Kunos et al. included three cases of CyberKnife treatment for recurrence of vulval epidermoid carcinoma following previous irradiation [17]. After positioning with gold markers, a dose of 24 Gy was delivered over three sessions of 8 Gy to the prescription isodose line of 70% to 75%. The previous doses received by the pelvis ranged from 45 to 74.6 Gy. The rate of local control at the treated targets was 100%, but unfortunately all the patients had recurrences outside of the irradiated fields. More recently, a series of 38 patients treated by CyberKnife to the pelvis was reported by the team of Muacevic et al [8]. The feasibility of this irradiation modality without placement of pelvic fiducial markers has been described as good. Seven patients had received previous pelvic irradiation, but no stratified analysis of this population was presented.

Other radiotherapy techniques have also been reported that show differing degrees of efficacy and toxicity. Brachytherapy allows the delivery of the prescribed dose to a well-defined volume. Reirradiation with brachytherapy in the treatment of uterine cervical cancer, either interstitially or more recently with high-dose rate (HDR) technique, peri-operatively or not, have been described [18-20]. In one series of 40 patients treated for carcinoma of the uterine cervix, reirradiation by means of interstitial brachytherapy was employed for 14 of the patients [21]. The rate of local control was 50% with a minimum post-treatment period of 2 years. Laparotomy was performed for all in the patient selection process. Gupta et al. reported a local control rate of 49% at three years in 15 patients that had previously received pelvic irradiation [22]. Charra et al. have treated 78 patients for carcinoma of the uterine cervix or endometrium [23]. They applied brachytherapy to the vaginal vault for recurrences in patients who were deemed eligible for this procedure. Thirty-seven percent of their patients had received radiotherapy during their initial treatment. The rate of local control at 5 years for these patients was 47% compared with 61% for patients without history of radiation treatment. Tolerance to this technique was good, but brachytherapy is an invasive technique requiring prolonged hospitalization and surgical intervention. Most importantly, this technique can only be recommended for recurrences in patients who are medically eligible and requires a rigorous patient selection.

On the other hand, there are a many studies about the treatment of recurrent adenocarcinoma of the rectum in an irradiated area. These are most often treated in a multimodal fashion, combining surgery with radiotherapy (external beam, perioperative radiotherapy), and chemotherapy. Tumor control rate at 3 years ranges from 14 to 56% [24-27]. The advantage of perioperative radiotherapy over other techniques has not been demonstrated. The recurrence rate in irradiated fields can be significant, described as up to 50% in the literature [28]. Intraoperative radiotherapy (IORT) has also been described [29-32]. IORT may stall recuperation from a macroscopically incomplete surgery [33]. One series from the Mayo Clinic including 51 patients that had received previous irradiation during their initial treatment showed a lower five-year survival rate among these patients with a history of previous irradiation compared to radiation-naïve patients [34]. The rate of local control and survival is greater when IORT is combined with neoadjuvant concomitant chemoradiotherapy and is only considered as part of a multimodality treatment regimen, surgery being at the center of the treatment despite associated difficulties. Other, less common treatments have been described and are still in development, such as hyperthermia [35-37].

One difficulty in our series was evaluation of local response to the treatment using the RECIST criteria. In fact, none of the patients demonstrated an unequivocal response to the treatment on comparative follow-up examinations. On the other hand, changes in the vascularization patterns of the tumors, and particularly, a decrease in uptake of contrast material were often reported by radiologists in charge of interpreting the imaging studies. We believe it is necessary to establish more suitable and specific criteria for monitoring these patients. Quality of life criteria are also important to take into account in the treatment of these patients, and they are insufficiently studied [38].

## Conclusions

Stereotactic radiotherapy by the robotic CyberKnife System can offer a brief and well-tolerated treatment for lateral pelvic recurrences in an area previously irradiated in non-operable patients otherwise not treatable. Long-term efficacy and toxicity need to be evaluated, however, the method is highly feasible. CyberKnife radiosurgery represents a new radiotherapeutic modality for patients with a history of previous pelvic irradiation. Additional study of treatment parameters and clinical outcomes including toxicity is planned.

## Competing interests

The authors declare that they have no competing interests.

## Authors' contributions

SD, JEB and XM conceived the study. SD and JEB collected data and drafted the manuscript. PN, BC, TL, HJ and EL participated in coordination and helped to draft the manuscript. SD performed the statistical analyses. EL provided mentorship and edited the manuscript. All authors have read and approved the final manuscript.
